# Virus-Specific Regulatory T Cells Ameliorate Encephalitis by Repressing Effector T Cell Functions from Priming to Effector Stages

**DOI:** 10.1371/journal.ppat.1004279

**Published:** 2014-08-07

**Authors:** Jingxian Zhao, Jincun Zhao, Stanley Perlman

**Affiliations:** Department of Microbiology, University of Iowa, Iowa City, Iowa, United States of America; Washington University, United States of America

## Abstract

Several studies have demonstrated the presence of pathogen-specific Foxp3^+^ CD4 regulatory T cells (Treg) in infected animals, but little is known about where and how these cells affect the effector T cell responses and whether they are more suppressive than bulk Treg populations. We recently showed the presence of both epitope M133-specific Tregs (M133 Treg) and conventional CD4 T cells (M133 Tconv) in the brains of mice with coronavirus-induced encephalitis. Here, we provide new insights into the interactions between pathogenic Tconv and Tregs responding to the same epitope. M133 Tregs inhibited the proliferation but not initial activation of M133 Tconv in draining lymph nodes (DLN). Further, M133 Tregs inhibited migration of M133 Tconv from the DLN. In addition, M133 Tregs diminished microglia activation and decreased the number and function of Tconv in the infected brain. Thus, virus-specific Tregs inhibited pathogenic CD4 T cell responses during priming and effector stages, particularly those recognizing cognate antigen, and decreased mortality and morbidity without affecting virus clearance. These cells are more suppressive than bulk Tregs and provide a targeted approach to ameliorating immunopathological disease in infectious settings.

## Author Summary

By repressing immune responses against pathogens, regulatory CD4 T cells are double edged swords. On one hand, they ameliorate immunopathological disease, diminishing morbidity but they also potentially contribute to pathogen persistence. Tregs have long been thought to be primarily directed at self-antigens, but we and others recently demonstrated the presence of pathogen-specific Tregs in infected animals. As is true for all pathogen-specific Tregs, few details are known about how these cells suppress T cell responses, especially those responding to the cognate epitope. Here, using mice with encephalitis caused by neurotropic coronavirus, we analyzed and compared the very earliest steps in the priming, proliferation and differentiation of Treg and Tconv responding to the same epitope, thereby providing new, fundamental information about these processes. Further, we identify a new role for pathogen epitope-specific Tregs in an acute infectious disease with an immunopathological component. Compared to bulk Tregs, they have the advantage of specifically diminishing numbers and function of pathogenic CD4 T cells responding to the same epitope without suppressing the anti-virus T cell response. Their use in the context of encephalitis or other infections would allow targeting of pathogenic CD4 T cell responses without generally suppressing the protective components of the immune response.

## Introduction

Regulatory T cells (Tregs), characterized by Foxp3 expression, have critical roles in suppressing pro-inflammatory immune responses, with ameliorating effects in autoimmune disease and deleterious consequences in the context of tumor clearance [1]. Tregs are also critical for the resolution of immune responses against pathogens. They are required for entry of immune cells into sites of inflammation in some viral infections [2], [3]. Additionally, in chronic viral infections, such as those caused by HIV, simian immunodeficiency virus, Friend virus and hepatitis C virus (HCV), Tregs contribute to pathogen persistence [4]. On the other hand, in acute viral infections caused by pathogens that include West Nile virus (WNV), herpes simplex virus (HSV) and mouse hepatitis virus (MHV), Tregs ameliorate acute disease [5]–[7]. If Tregs are depleted from mice infected with HSV or MHV, clinical disease is more severe [8], [9].

Until recently, Tregs were considered largely to recognize self antigens, but an increasing number of studies show that pathogen-specific Tregs are detected in infectious settings [10]–[14]. Further, these Tregs originate from thymus-derived pools, and are generally not generated by peripheral conversion from pathogen-specific effector CD4 T cell populations [10], [12], [13], [15], with the exception of Tregs specific for gut pathogens [16]. Studies of autoimmune diseases, such as diabetes mellitus, showed that adoptively transferred Tregs specific for an epitope at a site of inflammation were more suppressive than bulk populations of Tregs [17], [18]. Tregs specific for a *M. tuberculosis* (Mtb) CD4 T cell epitope are more suppressive than those that recognize a non-Mtb CD4 T cell epitope [19]. However, whether pathogen-specific Tregs are more potent than bulk populations of Tregs obtained from wild type mice has not been addressed in any infectious setting.

Mice infected with neurotropic strains of MHV develop acute encephalitis or acute and chronic demyelinating diseases [20]. Tregs are required to diminish immune-mediated disease in these mice. Thus, Treg depletion converted a nonlethal encephalitis to one with high mortality while transfer of bulk populations of Tregs to mice infected with a virulent strain of MHV prevented a lethal outcome [8]. In addition, transfer of naïve bulk populations of Tregs along with MHV-immune effector T cells to infected RAG1^−/−^ (Recombination Activation Gene1^−/−^) mice resulted in less severe clinical disease and diminished cell infiltration when compared to mice that received only effector T cells [7]. More recently, we identified Tregs that recognized the immunodominant CD4 T cell epitope (M133) in the brains of mice infected with the neuroattenuated rJ2.2 strain of MHV as well as in the T cell precursor pool of naïve mice [12]. Tregs at sites of inflammation adapt to the milieu by expressing transcription factors such as T-bet (Th1-type), STAT-3 (Th17-type) or IRF4 (Th2-type) [21] and as expected, brain-derived M133-specific Tregs in infected mice expressed T-bet. T-bet-mediated expression of CXCR3 is necessary for Treg migration to inflamed tissues [22]. These cells expressed cytokines such as IFN-γ and TNF, in addition to IL-10 when stimulated with M133 peptide directly *ex vivo*. Further, we showed that IFN-γ production was not an *in vitro* phenomenon since IFN-γ expression by Tregs was detected directly *ex vivo* in the absence of peptide stimulation if mice were treated with brefeldin A prior to sacrifice [12].

As is true for all pathogen-specific Tregs, few details are known about how these cells affect T cell responses, especially those responding to the cognate epitope or whether these cells are more immunosuppressive than bulk Tregs. Addressing these questions directly in wild type mice is difficult because M133-specific Tregs comprise only a small fraction of total Tregs. To circumvent this problem, we developed a mouse transgenic for the expression of an M133-specific T cell receptor [23] and used Tregs (M133 Tregs) from these mice in the current study. The results show that Tregs function both in the draining lymph nodes (deep cervical lymph nodes, DCLN, and to a lesser extent, superficial cervical lymph nodes, CLN) and in the brain to ameliorate encephalitis severity.

## Results

### Preferential recruitment of M133-specific Tregs to the rJ2.2-infected brain

**Figure 1:**
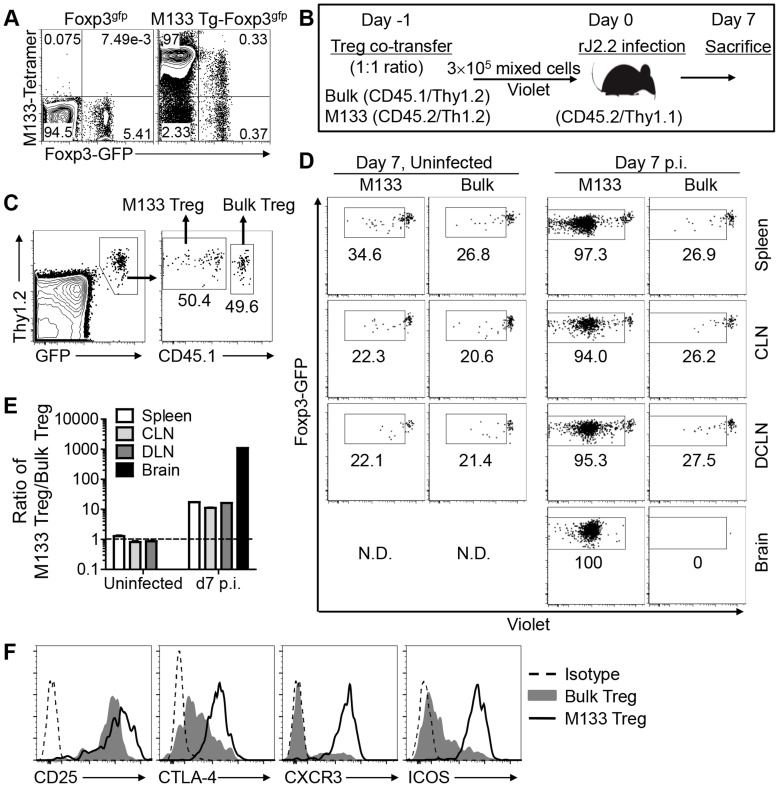
M133 Tregs, but not bulk Tregs proliferate and are recruited to the brains of rJ2.2-infected mice. (**A**) Blood was obtained from B6-Foxp3^gfp^ and M133 Tg-Foxp3^gfp^ mice and analyzed for presence of M133-specific Tregs and Tconv using I-A^b^/M133 tetramers. Representative plots after gating on CD4 T cells are shown. (**B**) Experimental design. Bulk and M133 Tregs were purified by flow cytometry from the spleen and LNs of Foxp3^gfp^ and M133 Tg-Foxp3^gfp^ mice, respectively, mixed at a 1∶1 ratio and labeled with Violet. A total of 3×10^5^ cells were transferred to CD45/Thy1 congenic mice one day prior to infection with rJ2.2. (**C**) Gating strategy for identification of M133 and bulk Tregs in recipient mice is shown. (**D**) Representative plots showing proliferation of cells in the indicated tissues at day 7 p.i. The box in each panel indicates cells that have proliferated. N.D., not detectable. Percentages of divided cells are shown. (**E**) The ratio of M133 Treg/bulk Treg is shown. The data are representative of three independent experiments (**A, C, D**) or pooled from three experiments (**E**). (**F**) 2×10^5^ M133 or Bulk Tregs were transferred to Thy1.1 mismatched mice and expression levels of indicated markers on transferred cells in the DCLN at day 4 p.i. are shown. To analyze bulk Tregs, cells from 5 recipient mice were pooled. Data are representative of analyses of three individual recipients (M133 Tregs) and 2 pooled samples (bulk Tregs).

In the natural infection, M133-specific Tregs were detected in the rJ2.2-infected brain [12]. However, adoptively transferred bulk populations of splenic C57BL/6 (B6) Tregs (bulk Tregs) were minimally detected in the infected central nervous system (CNS) [7]. To reconcile these disparate results, we co-transferred bulk Tregs and M133 Tregs derived from uninfected Foxp3^gfp^ and M133 Tg-Foxp3^gfp^ mice, respectively. Approximately 0.5–1% of the CD4 T cells in the blood of M133 Tg mice expressed Foxp3; 50% of Foxp3^+^ and >97% of Foxp3^−^CD4 T cells (Tconv) bound I-A^b^/M133 tetramer (**Figure 1A**). Equal numbers of Violet-labeled bulk and M133 Tregs were transferred to B6 mice, some of which were infected with rJ2.2 24 hours later (**Figure 1B**). Tregs were identified in the lymphoid tissues of infected and uninfected recipient mice seven days after infection, using the gating strategy shown in **Figure 1C**. Equal numbers of bulk and M133 Tregs were detected in the spleen, cervical and deep cervical lymph nodes (CLN, DCLN) of uninfected mice and neither population proliferated substantially. In contrast, after infection, M133 but not bulk Tregs proliferated extensively in all three peripheral lymphoid tissues. Further, only M133 Tregs entered the infected brain to a detectable extent (**Figure 1D**). By day 7 post infection (p.i.), the ratio of M133 to bulk Tregs ranged from 10∶1 in lymphoid tissue to greater than 1000∶1 in the brain (**Figure 1E**). In addition to exhibiting less proliferation, transferred bulk Tregs in the DCLN were less activated when assessed by measuring expression of CD25, CTLA-4, CXCR3 and ICOS at day 4 p.i. (**Figure 1F**).

### Initial proliferation of M133-specific Tconv and Tregs occurs in the DCLN

**Figure 2:**
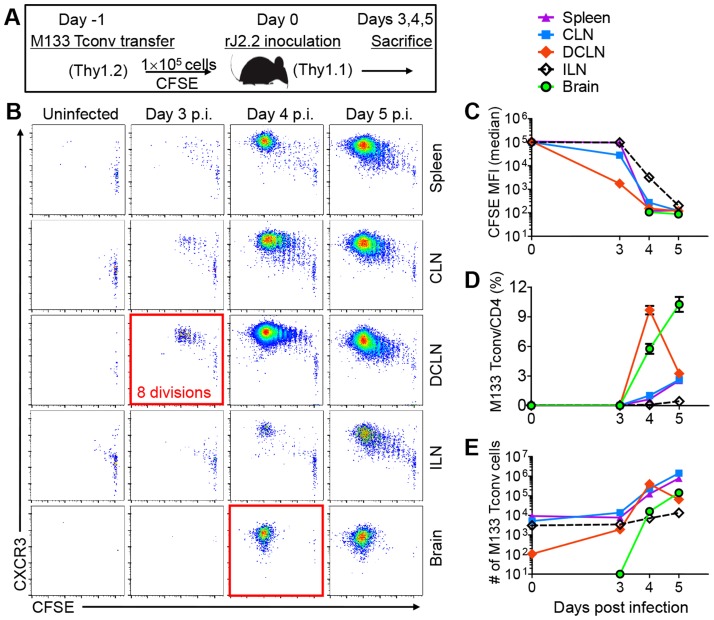
Initial proliferation of M133 Tconv occurs in the DCLN. (**A**) Experimental design. 1×10^5^ CFSE labeled M133 Tconv (Treg-depleted CD4 T cells) were transferred to Thy1 congenic mice one day prior to rJ2.2 infection. (**B**) Representative plots showing proliferation of transferred cells and CXCR3 expression. (**C**) CFSE levels on transferred cells at several times p.i. MFI, mean fluorescence intensity. (**D, E**) Frequency (**D**) and numbers (**E**) of M133 Tconv at the indicated times after infection. The data are representative of four independent experiments with 3 mice per time point in each.

To identify the site of initial priming and expansion of M133 Tregs compared to Tconv, we first analyzed priming of M133 Tconv (Treg-depleted CD4 T cells) by transferring CFSE-labeled cells one day prior to infection (**Figure 2A**). Mice were sacrificed at days 3, 4 and 5 p.i. and analyzed for CFSE dilution (indicative of cell division) and for CXCR3 expression (required for T cell migration to the inflamed brain [24]) by M133 Tconv in the spleen, CLN, DCLN, inguinal lymph nodes (ILN) and brain. CFSE dilution occurred initially in the DCLN (**Figure 2, B and C**). Expression of CXCR3 was upregulated within 1–2 rounds of proliferation (**Figure 2B**). By day 4 p.i., M133 Tconv accounted for 10% of all CD4 T cells in the DCLN, a frequency higher than in any other lymphoid tissues that were examined (**Figure 2D**). While the numbers of M133 Tconvs decreased dramatically between days 4 and 5 p.i. in the DCLN (**Figure 2E**), they continued to increase in other lymphoid tissues. These results suggested that M133 Tconvs were primed in the DCLN and migrated to other sites. By day 5, cells that had undergone varying numbers of divisions were detected in all tissues except the brain, where only highly divided cells (complete loss of CFSE labeling) were present.

**Figure 3:**
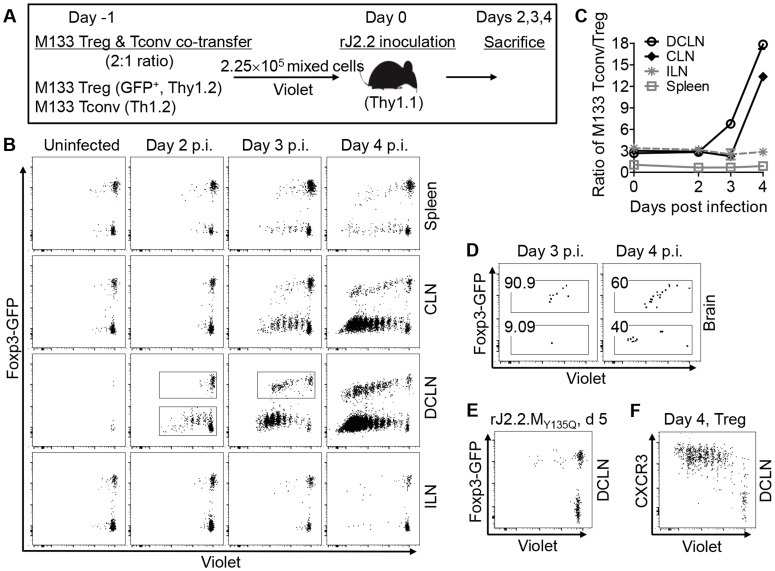
Initial proliferation of M133 Tregs in the DCLN is delayed compared to M133 Tconv. (**A**) Experimental design. M133 Tregs and M133 Tconv (Treg-depleted CD4 T cells) were mixed at a 2∶1 ratio and labeled with Violet. 2.25×10^5^ cells were transferred to Thy1 congenic mice one day prior to infection with rJ2.2. (**B**) Representative plots showing proliferation of M133 Tconv and Treg. (**C**) Ratio of M133 Tconv/Treg in several organs at various times p.i. (**D**) Dot plot showing entry of M133 Treg and Tconv into the infected brain at early times p.i. Percentages of M133 Treg and Tconv after gating on transferred cells are shown. (**E**) Representative plot showing proliferation of M133 Tconv and Treg in mice infected with rJ2.2.M_Y135Q_, which lacks epitope M133 expression, at day 5 p.i. (**F**) Representative plot showing CXCR3 expression by M133 Treg as they proliferate. The data are representative of four (**B, C, D, F**, three mice per time point) or one (**E**, four individual mice) independent experiments.

To confirm the DCLN as the site of proliferation of Tconv and determine whether Tregs were also primed at this site, we transferred Violet-labeled M133-specific Tconv and Tregs in a 1∶2 ratio to mice one day prior to infection (**Figure 3A**). This resulted in a 1∶1 ratio because only 50% of Tregs were M133-specific (**Figure 1A**). Cells were analyzed at days 2–4 instead of days 3–5 to capture early cell proliferation, since we had observed substantial proliferation of Tconv in the DCLN by day 3 p.i. when only Tconv were transferred (**Figure 2B**). By day 2 p.i, we observed five generations (4 divisions) of Violet-labeled Tconv in the DCLN but not other tissues. In contrast, Treg proliferation was not detected until day 3 p.i. with Violet dilution occurring initially in the DCLN (**Figure 3B**). By day 4 p.i., M133 Tconv and Tregs that had undergone extensive division were detected in the DCLN and CLN, with Tconv proliferation occurring to a greater extent than that of Tregs. This resulted in a dramatic increase in the ratio of Tconv to Tregs from day 3 to 4 p.i. in the DCLN and CLN (**Figure 3C**). Of note, only very few M133 CD4 T cells entered the brain at days 3 or 4 p.i., but remarkably, the majority of these cells were Tregs (**Figure 3D**). Tregs were able to enter the brain after fewer cycles of proliferation when compared to Tconv (**Figure 3D** **and** **4B**). Exposure to M133 antigen was required for proliferation because neither M133 Tconv nor M133 Treg proliferated in mice infected with a rJ2.2 mutant (rJ2.2.M_Y135Q_) in which epitope M133 expression was abrogated (**Figure 3E**). Similar to M133 Tconv, we detected CXCR3 expression on M133 Tregs within 1–2 generations of division (**Figure 3F**).

**Figure 4:**
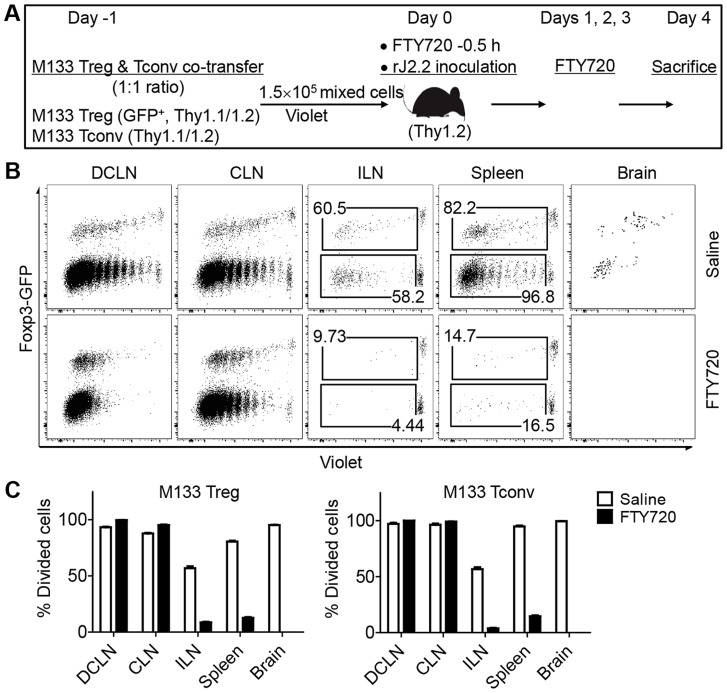
M133 Tconv and Treg proliferation occurs in DCLN and CLN after FTY720 treatment. (**A**) Experimental design. M133 Tregs and M133 Tconv were mixed at a 1∶1 ratio and labeled with Violet. 1.5×10^5^ cells were transferred to Thy1 congenic mice one day prior to infection with rJ2.2. Mice were treated with FTY720 or saline 0.5 hour prior to infection and on days 1, 2, 3 p.i. (**B**) Representative plots showing Violet dilution at day 4 p.i. Numbers are percentage of divided Tregs (upper) or Tconv (lower). (C) Summary of data from three independent experiments with three mice in each.

While these results showed that the DCLN were the initial site of priming, M133 Tconv and Treg proliferation was also detected in the spleen and inguinal lymph nodes. To distinguish priming at these distal sites and migration after initial priming in the DCLN, we treated mice with FTY720, a drug which inhibits cell egress from lymph nodes [25]. In these experiments, equal numbers of M133 Tconv and Tregs were transferred to B6 mice and mice were treated with the drug 0.5 hr prior to infection and then on a daily basis (**Figure 4A**). Proliferating M133 Tconv or Tregs were barely detectable in the spleen or ILN after FTY720 treatment at day 4 p.i., but proliferation continued unabated in the DCLN and to a lesser extent, in the CLN. No transferred cells were detected in the brain after drug treatment (**Figure 4, B and C**). Further, only highly divided cells were detected in the DCLN in treated mice whereas in the absence of FTY720, cells with only partial dye dilution were detected, suggesting that the latter were newly recruited. Overall, these data demonstrate that all priming occurs in the cervical lymph nodes, predominantly in the DCLN.

### M133 Tregs inhibit M133 Tconv proliferation in, and egress from, the DCLN

**Figure 5:**
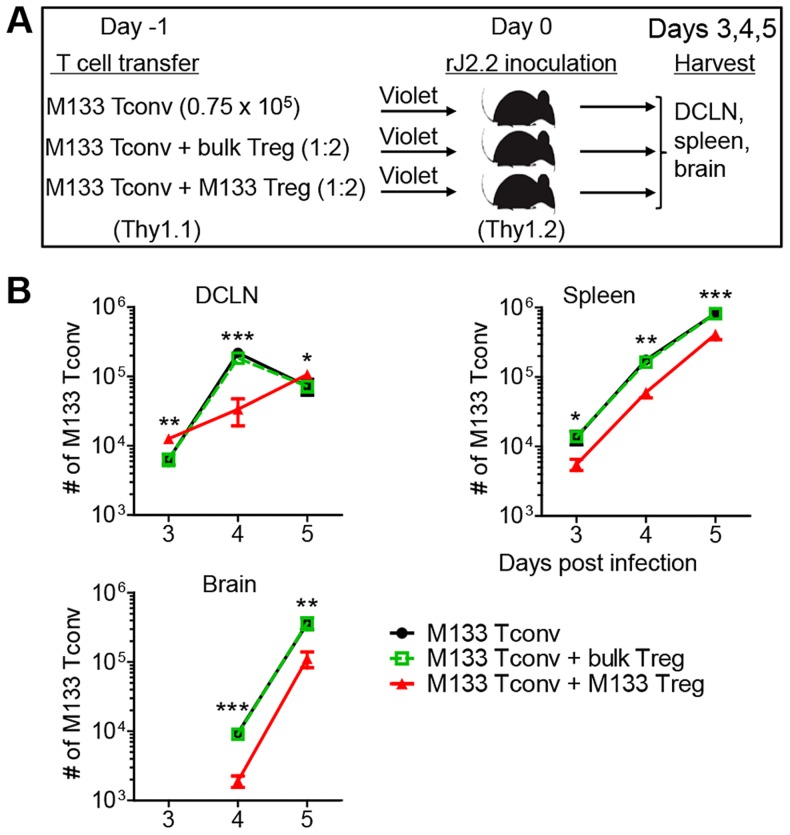
M133 Tregs modulate M133 Tconv accumulation in the DCLN, spleen and brain. (**A**) Experimental design. M133 Tconv alone or a mixture of M133 Tconv and bulk Tregs or M133 Tconv and M133 Tregs (1∶2 ratio) were labeled with Violet. After labeling, 0.75×10^5^ M133 Tconv or 2.25×10^5^ mixed cells were transferred into Thy1 congenic mice one day prior to rJ2.2 infection. (**B**) Numbers of M133 Tconv in DCLN, spleen and brain of recipient mice at the indicated times p.i. The data in B are representative of 3 independent experiments with 3–4 mice/time point. Asterisks indicate statistical significance when mice receiving M133 Tconv and M133 Tregs were compared to those receiving only M133 Tconv or M133 Tconv and bulk Tregs. *P<0.05, **P<0.01, ***P<0.001.

Since M133 Tregs and Tconv expanded at the same site, we reasoned that M133 Tregs would suppress Tconv activation and proliferation if both were present in the DCLN, and that M133 Tregs would be more suppressive than bulk Tregs since the latter did not proliferate. To examine these possibilities, we transferred M133 Tconv in the presence or absence of co-transferred M133 or bulk Tregs and sacrificed them at days 3–5 p.i. (**Figure 5A**). Bulk Tregs had no effect on numbers of M133 Tconv in the DCLN, spleen or brain at any time (**Figure 5B**). In contrast, the presence of M133 Tregs resulted in decreased numbers of Tconv in the spleen and brain at all times p.i., suggesting an effect on Tconv proliferation. However, the effects of transferred M133 Tregs were more complicated in the DCLN. M133 Tconv numbers in the DCLN were increased at day 3 and 5 p.i., but decreased at day 4.

**Figure 6:**
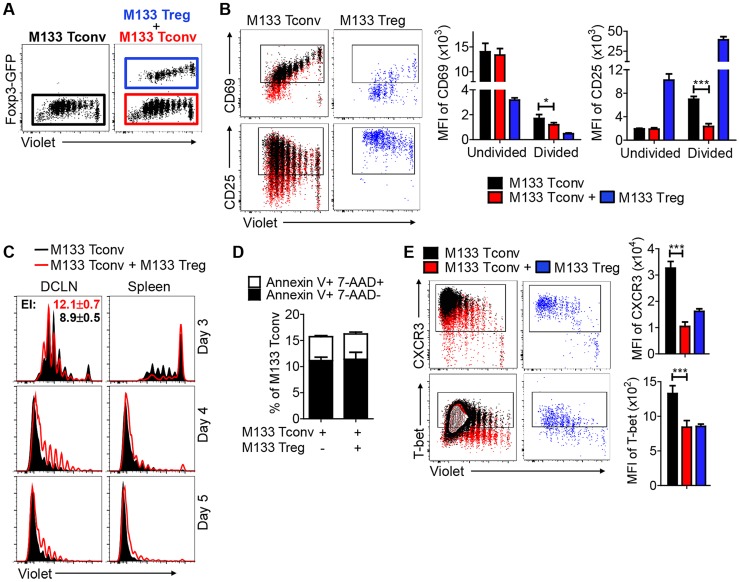
M133 Tregs inhibit M133 Tconv proliferation in, and egress from, the DCLN. M133 Tconv or M133 Tconv and M133 Tregs (1∶2 ratio) were labeled with Violet and transferred into Thy1 congenic mice prior to rJ2.2 infection as described in Figure 5A. Organs were harvested at the indicated time points and lymphocyte populations examined directly *ex vivo*. (**A**) Representative plots showing gating of transferred M133 Tconv (GFP^−^) in the absence (**black**) or presence of (**red**) co-transferred M133 Tregs, and of M133 Tregs (GFP^+^, **blue**). These gates and colors were applied in **B–E**. (**B**) Representative dot plots show CD69 and CD25 expression on M133 Tconv and M133 Tregs in DCLN at day 3 p.i. Boxed cells are positive for CD69 or CD25 expression. Summary data show expression levels of CD69 and CD25 on undivided and divided M133 Tconv. (**C**) Overlayed histograms showing proliferation of M133 Tconv in the absence or presence of co-transferred M133 Tregs in DCLN and spleen at days 3, 4 and 5 p.i. Expansion index (EI) of M133 Tconv was calculated using Flowjo software. (**D**) Apoptosis of M133 Tconv in the DCLN at day 4 p.i. was analyzed by Annexin V/7-AAD staining. (**E**) CXCR3 and Tbet levels on M133 Tconv and M133 Tregs in DCLN at day 4 p.i. The data in B-E are representative of 2–6 independent experiments with 3–5 mice/time point. *P<0.05, ***P<0.001.

To probe this in more detail, M133 Tconv activation and proliferation, based on Violet dilution, was analyzed with or without co-transferred M133 Tregs (**Figure 6**). M133 Tconv were gated as shown in **Figure 6A**. Activation was assessed by measuring levels of CD25 and CD69 on Tconv in the DCLN at day 3 p.i. (**Figure 6B**). Equivalent levels of CD25 and CD69 were detected on undivided M133 Tconv. However, even though expression of CD25 and CD69 followed similar kinetics in the presence or absence of Tregs, levels of both molecules were lower in the presence of Tregs after cells began to divide. These results suggest that the initial activation of M133 Tconv was not affected by the presence of Tregs, perhaps because of the lag in Treg relative to Tconv activation, shown in Figure 3. However, as M133 Tregs became activated and proliferated, they functioned to downregulate both molecules on Tconv. Of note, CD69 levels were lower on M133 Tregs than on M133 Tconv in mice that received both types of cells, while, as expected, CD25 levels were higher on M133 Tregs (**Figure 6B**, blue bars).

M133 Tregs inhibited M133 Tconv proliferation in the DCLN at days 4 and 5 p.i., when cells were examined directly *ex vivo*. The effect of Tregs on proliferation is most likely even greater than shown in the figure because a limitation of this assay is that dye dilution cannot be detected beyond 9 divisions. Remarkably, at day 3 p.i., Tconv that had undergone more divisions were detected in the presence of Tregs, resulting in a higher expansion index (EI) (**Figure 6C**). Of note, only the EI in the DCLN at day 3 p.i. is shown because the EI cannot be calculated when there are too few cells in the parent generation (DCLN and spleen at days 4 and 5 p.i.) or too few proliferated cells (spleen at day 3 in the presence of M133 Tregs). Further, Tconv numbers in the DCLN decreased from day 4 to day 5 when transferred alone, indicating significant cell egress, while numbers of these cells continued to increase in the presence of M133 Tregs (**Fig. 5B**). These results, in conjunction with decreased Tconv numbers in the spleen and brain at all times in the presence of M133 Tregs (**Figure 5B** **and** **6C**), suggest that Tregs inhibited egress from the DCLN in addition to effects on proliferation. Of note, decreased Tconv numbers could reflect increased levels of apoptosis, but the fraction of Tconv apoptosis was the same in the presence or absence of transferred M133 Tregs in the DCLN at day 4 (**Figure 6D**).

In addition, levels of CXCR3 on Tconv in the DCLN were much lower at day 4 when Tregs were co-transferred (**Figure 6E**). Since T cell entry into sites of inflammation depends in part on CXCR3 expression, this decreased expression contributed to decreased numbers in the brain and perhaps also in the spleen. CXCR3 transcription is regulated by T-bet [26]; consistent with this, T-bet expression in Tconv was less elevated in the presence of transferred Tregs (**Figure 6E**). Thus, in the DCLN, M133 Tconv accumulation was determined by a balance of opposing effects on migration and proliferation, while in the spleen and brain, both effects contributed to decreased numbers of Tconv. Notably, CXCR3 and T-bet levels on M133 Tregs and M133 Tconv were similar when these cells were co-transferred (Figure 6E, blue bars).

### Differential kinetics of T-bet and cytokine expression by M133 Tconv and Tregs in DCLN

**Figure 7:**
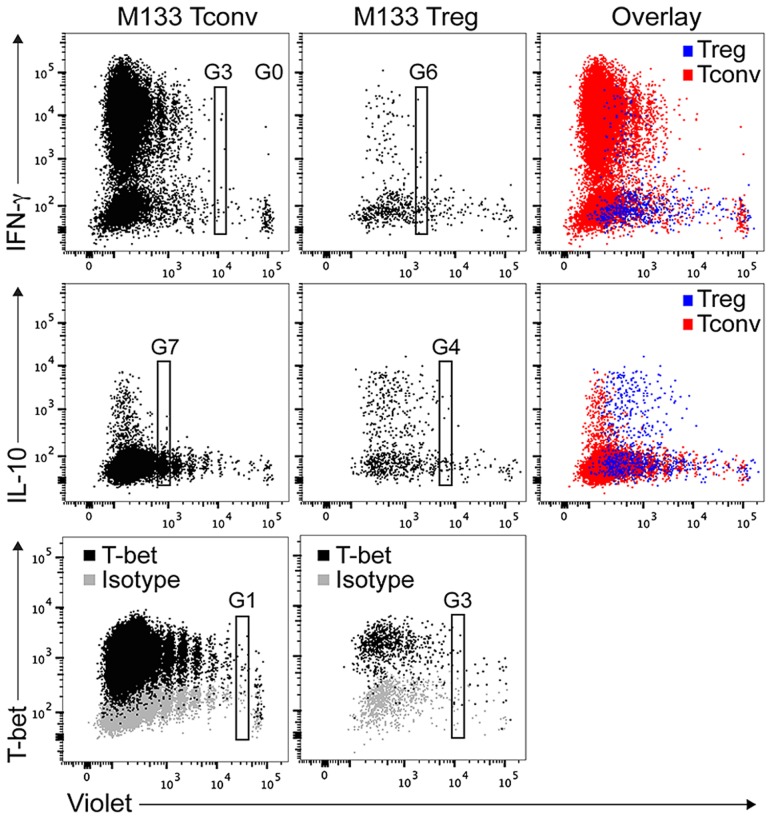
M133 Tconv and Tregs exhibit differential kinetics of T-bet and cytokine expression in DCLN. Violet labeled M133 Tconv (0.75×10^5^) or M133 Tregs (1.5×10^5^) were transferred to Thy1 congenic mice one day prior to rJ2.2 infection. DCLN cells were pooled from six recipient mice at day 3.5 after infection. Cells were examined for T-bet expression directly *ex vivo* or for IFN-γ and IL-10 expression after M133 peptide stimulation. G0: generation 0, G1: generation 1, etc. Dot plots show T-bet and cytokine expression by M133 Tconv and Tregs as they proliferate. Data are representative of two independent experiments.

M133-specific Tregs in the infected brain express T-bet and several cytokines (IFN-γ, TNF and IL-10) [12]. We next sought to determine whether this differentiation to a Th1 phenotype occurred after Tregs had entered the brain, or earlier in the DCLN. We transferred either M133 Tconv or Treg into mice prior to rJ2.2 infection and measured T-bet and cytokine expression in the DCLN at day 3.5 p.i. (**Figure 7**). T-bet was expressed by both types of cells at this early time post infection, after either one (Tconv) or three (Treg) cell divisions. Further, IFN-γ and IL-10 were expressed by both M133 Tconv and Tregs in the DCLN after direct *ex vivo* stimulation with M133 peptide. Tregs required more cell divisions to express IFN-γ than Tconv but conversely, IL-10 expression by Tregs occurred after fewer cell divisions than Tconv. Thus, Treg ability to express cytokines did not require entry into the infected brain, but rather the milieu in the draining DCLN was sufficient to trigger differentiation. These cells then migrated to the infected brain, already competent for cytokine expression.

### Transferred M133 Tregs enhance survival and diminish the M133 Tconv immune response in the infected brain

**Figure 8:**
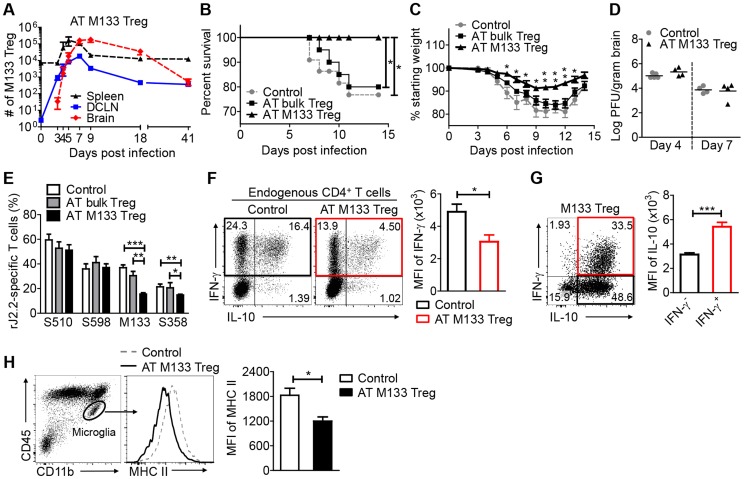
Transferred M133 Tregs enhance survival and diminish the M133 Tconv immune response in the brain. 10^5^ M133 Tregs, bulk Tregs or B6-derived Foxp3^−^CD4 T cells (control group) were transferred to Thy1 congenic mice one day prior to rJ2.2 infection. (**A**) M133 Treg numbers in spleen, DCLN and brain of recipient mice from days 0 to 41 p.i. (**B–D**) Survival (**B**), weight loss (**C**) and viral titers in the brains (**D**) of recipient mice were monitored. 18–22 mice in 4 independent experiments were analyzed for survival and weight loss. In (**C**), *P<0.05, **P<0.01 weights of M133 Treg recipients compared to mice that received bulk Tregs or control cells; (**E–G**) Lymphocytes were prepared from brains of recipient mice at day 7 p.i. and stimulated with the indicated peptides. (**E**) Frequencies of S510 and S598-specific cells within the CD8 T cell population and M133- and S358-specific cells within the CD4 T cell population are shown. 6–10 mice in 3–5 independent experiments were analyzed. (**F**) Brain-derived lymphocytes were harvested from mice that received control cells or M133 Tregs. Representative dot plots show IFN-γ and IL-10 expression by endogenous CD4 T cells after M133 peptide stimulation. Summary data show expression levels of IFN-γ by M133-specific CD4 T cells in the absence (**black**) or presence (**red**) of M133 Treg. (**G**) Representative dot plots (from the same sample as in **F** (right hand dot plot)) show IFN-γ and IL-10 expression by exogenous M133 Tregs. Note that lower levels of IFN-γ were expressed by M133 Tregs when compared to Tconv. Summary data show expression levels of IL-10 by IFN-γ^-^ (**black**) and IFN-γ^+^ M133 Tregs (**red**). Data in (**F**) and (**G**) are representative of three independent experiments with at least 3 mice/group. (**H**) Expression levels of MHC II on brain microglia at day 7 p.i. Data are representative of three independent experiments with 3–5 mice/group. *P<0.05, **P<0.01, ***P<0.001.

To examine the effects of transferred M133 Tregs on clinical disease, we transferred 10^5^ M133 Tregs (in the absence of M133 Tconv), bulk Tregs or bulk Tconv (control group) to infected mice one day prior to rJ2.2 infection. Consistent with priming in the DCLN, numbers of M133 Tregs increased dramatically from day 0 to day 3 p.i. in the DCLN but not the spleen. M133 Tregs were first detected in the brain at day 3, peaked at day 7 in both the brain and DCLN and then gradually declined in both organs as the infection resolved (**Figure 8A**). Transferred M133 Tregs, but not bulk Tregs improved survival and diminished weight loss (**Figure 8, B and C**) without affecting virus clearance (**Figure 8D**). M133 Tregs decreased the frequencies of M133-specific CD4 T cells in the brain (**Figure 8E**). In addition, transferred M133 Tregs inhibited the effector function of these cells, manifested by reduced IFN-γ expression per cell (**Figure 8F**). The transferred Tregs also diminished the frequency of CD4 T cells recognizing the subdominant CD4 T cell epitope (epitope S358) but did not change the CD8 T cell response to the immunodominant S510 and subdominant S598 epitopes (**Figure 8E**).

As expected, most of these transferred M133 Tregs expressed IL-10 or IL-10 and IFN-γ after direct *ex vivo* peptide stimulation (**Figure 8G**). IFN-γ was expressed at lower levels per Treg than per effector CD4 T cell (compare **Figure 8** **, F** and **G**). Of note, IFN-γ^+^ M133 Tregs expressed IL-10 at higher levels than those not expressing IFN-γ (**Figure 8G**) perhaps reflecting the expression of IFN-γ only after cells had extensively proliferated and were presumably highly activated (**Figure 7**).

To provide further support for the notion that M133 Tregs suppressed immune function in the CNS in addition to the DCLN, we assessed microglia activation after infection by measuring levels of MHC class II. Transfer of M133 Tregs resulted in decreased activation of microglia, as shown by diminished expression of MHC class II (**Figure 8H**) at day 7 p.i. Overall, these data indicate that M133 Tregs functioned in both the draining lymph nodes and site of infection, to diminish the effects of pathogenic CD4 T cells.

**Figure 9:**
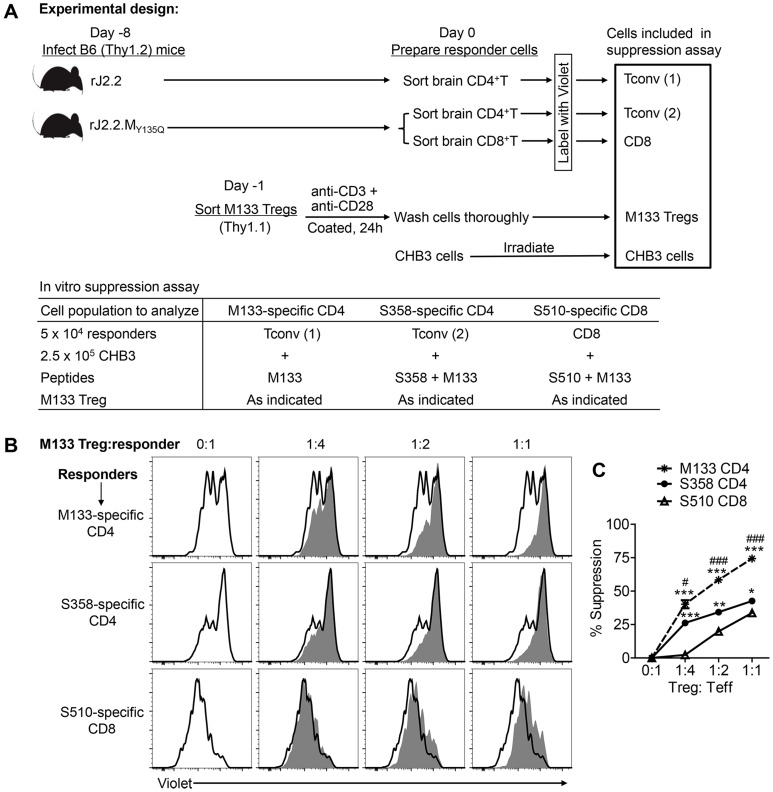
M133 Tregs preferentially inhibit proliferation of M133-specific Tconvs in an *in vitro* suppression assay. (**A**) Experimental design. M133, S358 and S510-specific T cells were obtained from infected mice, labeled as described in Materials and Methods and used as responder cells. M133-specific Tregs were pre-activated *in vitro* for 24 hours and co-cultured with the labeled responders and irradiated antigen presenting cells (CHB3 cells). Cells were stimulated with the indicated peptides for 66 hours and then analyzed for Violet dilution. (**B**) Representative histograms showing proliferation of responders in the absence (open) or presence (shaded) of M133 Tregs. (**C**) Summary of data from 3 rJ2.2 and 3 rJ2.2.M_Y135Q_-infected mice. *P<0.05, **P<0.01, ***P<0.001, M133 or S358 suppression compared to S510 suppression; ^#^ *P*<0.05, ^###^ *P*<0.001, M133 suppression compared to S358 suppression.

To further buttress the conclusion that M133 Tregs preferentially suppressed the M133 Tconv response, we performed an *in vitro* suppression assay (**Figure 9**). Because S358 and S510 TCR Tg mice were not available, we obtained CD4 and CD8 T cells from infected mice and labeled them with Violet. Since these cells had been activated *in vivo*, M133 Tregs, isolated from uninfected M133 TCR Tg mice were therefore pre-activated *in vitro* with anti-CD3 and anti-CD28 antibodies. In all cases, M133 peptide was included in the *in vitro* suppression assay to activate M133 Tregs. Consequently, in order to distinguish proliferation of M133 and S358 CD4 or S510 CD8 T cells, the latter cells were obtained from mice infected with virus lacking expression of the M133 epitope (rJ2.2.M_Y135Q_). As shown in Figure 9B and C, M133 Tregs preferentially suppressed the proliferation of M133 Tconv. In agreement with the results shown in Figure 8E and our previous results [27], M133 Tregs inhibited the proliferation of S358-specific CD4 T cells more potently than that of S510-specific CD8 T cells.

## Discussion

Tregs specific for epitopes present at sites of autoimmune inflammation diminish disease more effectively than bulk Treg populations [28]. Here we extend these results to mice with coronavirus-induced encephalitis and show that Tregs specific for a virus-specific CD4 T cell epitope are highly activated and protective. M133 Tregs proliferate, express cytokines, particularly the immunosuppressive cytokine IL-10, and suppress M133 Tconv proliferation in the DCLN. Further, they inhibit Tconv migration from the site of priming, the DCLN. After transfer to rJ2.2-infected mice, M133 Tregs increase survival and decrease weight loss when compared to bulk populations of Tregs and decrease numbers and effector function of virus-specific CD4 T cells in the brain. Many of these effects reflect Treg function in the DCLN, but additionally, microglia activation in the brain was diminished after M133 Treg transfer. We showed previously that large numbers of bulk Tregs could tamper the immune response in the infected brain, by causing a dimunition in total numbers of infiltrating cells, without affecting the cellular composition [7]. However, the use of virus epitope-specific Tregs allowed for a targeted immune response with effects primarily on T cells responding to the same epitope, which are pathogenic in this case. It should be noted that treatment with pathogen-specific Tregs is not always beneficial. In mice infected with Mtb, adoptive transfer of pathogen epitope-specific Tregs resulted in delayed effector CD4 and CD8 T cell responses to non-cognate Mtb epitopes in the draining lymph nodes and lungs and subsequent increased bacterial load [19].

Virus-specific Tregs were primed in the same draining lymph nodes as effector T cells, but the kinetics of proliferation and cytokine production were different. The onset of Treg proliferation lagged by approximately one day, possibly because Treg proliferation was dependent upon IL-2 production by Tconv in the DCLN. This lag resulted in a lack of suppression of initial Tconv activation. However, even by 1–2 divisions, Tconv downregulated CD69 and CD25 expression in the presence of M133 Tregs (**Figure 6B**), suggesting decreased activation compared to mice that received only M133 Tconv. Notably, even after Treg proliferation began, the M133 Tconv/Treg ratio continued to increase in the DCLN and CLN, the sites of priming, but not the spleen or ILN (**Figure 3C**), suggesting preferential retention or proliferation and perhaps, recruitment of Tconv. Preferential M133-specific effector CD4 T cell expansion also occurs in the natural infection because the ratio of M133-specific Tregs/T effector cells in the naïve precursor pool is 0.15 but this decreases to 0.02 in the infected brain [12].

It is noteworthy that M133 Tconv proliferation appeared to increase in the DCLN at day 3 p.i. in the presence of co-transferred M133 Tregs. However, rather than enhancing proliferation at this time, we postulate that Tregs actually function to inhibit Tconv egress from the DCLN, resulting in the retention of proliferated M133 T conv. Consistent with this, fewer M133 Tconv were detected in the spleen at this time point after co-transfer (**Figure 5B**). These results are similar to those obtained from FTY720-treated mice (**Figure 4**), which showed that when T cell egress was blocked, proliferating cells accumulated in the draining lymph nodes but were not detected in distal lymphoid tissues. Tconv activation in the DCLN was also diminished in the presence of transferred M133 Tregs (**Figure 6B**). Tregs use a multitude of mechanisms to suppress Tconv and dendritic cell function, including the production of IL-10 [1], [29]. IL-10 was expressed by M133 Tregs within 4 divisions and likely contributed to the suppression of Tconv proliferation in the DCLN (**Figure 7**). Collectively, these results suggest that the relative numbers of virus-specific Tconv in the DCLN, spleen and brain of mice that received M133 Tregs, compared to those did not, reflect a complicated interplay between Treg effects on Tconv activation, proliferation and migration.

In contrast to these results, Tregs were required for optimal migration of inflammatory cells including dendritic cells to the site of infection in mice infected with genital HSV, lymphocytic choriomeningitis virus or respiratory syncytial virus. In their absence, virus clearance was delayed and survival decreased [2], [3]. In these studies, Tregs were depleted prior to infection, a time when very few Tregs are virus-specific [12], [15]. Together, the results from these studies and the present one suggest that non virus-specific Tregs are important for initial egress from lymphoid tissue to sites of inflammation, but virus-specific Tregs, which would be induced later in the infection, function to inhibit migration of virus-specific T cells to the infected site.

M133 Tregs also decreased the numbers of co-transferred M133 Tconv in the infected brain (**Figure 5B**). M133 Tregs, if transferred alone, suppressed the endogenous M133 and, to a lesser extent, S358-specific CD4 T cell responses (**Figure 8E**). Similar results were obtained when the suppressive ability of M133 Tregs was examined *in vitro* (**Figure 9**). Preferential suppression of the M133 Tconv compared to the S358 Tconv response may reflect antigen competition between endogenous M133 Tconv and transferred M133 Tregs. This suppressive effect was not generalized since the number and frequency of cells responding to CD8 T cell epitopes S510 and S598 in mice were not changed by the presence of M133 Tregs (**Figure 8E**) and the proliferation of S510-specific CD8 T cells *in vitro* was less inhibited by M133 Tregs (**Figure 9**). This lack of effect on CD8 T cell responses in the brain may explain why virus clearance is not changed in the presence of M133 Tregs (**Figure 8D**); virus clearance is largely CD8 T cell dependent [20]. The diminished numbers of M133-specific Tconv reflected decreased proliferation in the DCLN, but also likely resulted from decreased expression of CXCR3 on M133 Tconv when M133 Tregs were co-transferred (**Figure 6E**). Treg-mediated downregulation of CXCR3 expression on Tconv in draining lymph nodes was also observed in mice with autoimmune diabetes [30]. Further, transfer of M133 Tregs resulted in decreased expression of MHC class II on microglia, which potentially diminished their ability to activate T cells (**Figure 8I**). In addition, M133 Tregs entered the brain at earlier times than M133 Tconv (Figure 3D). Together, these results suggest that Tregs directly suppressed microglia activation, but it is also possible that decreased numbers of Tconv in the brain contributed to lower microglial MHC class II expression.

Tregs in the infected brain expressed IFN-γ, albeit at lower levels on a per cell basis when compared to Tconv (**Figure 8, F and G**). We and others have shown that the expression of IFN-γ does not abrogate the immunosuppressive functions of Tregs *in vitro* or in mice and is even required for optimal activity in some settings [12], [27], [31], [32]. However, Treg-expressed IFN-γ may contribute to the inflammatory milieu, although whether this is physiologically significant will require further work. It is also notable that while Tregs in the brains of rJ2.2-infected mice expressed IFN-γ, IFN-γ expression by Tregs has not been detected in the lungs or draining lymph nodes of mice infected with Mtb or influenza A virus [10], [13]. Although M133 Tregs ameliorated clinical disease in rJ2.2-infected mice, their immunosuppressive efficacy in the inflamed brain may have been suboptimal. In mice with experimental autoimmune encephalomyelitis, myelin-specific Tregs were detected in the brain and spinal cord, but their function was inhibited by TNF and IL-6 [33]; these two cytokines are expressed in the rJ2.2-infected CNS [20], [34].

Transfer of M133 compared to bulk Tregs resulted in improved outcomes (**Figure 8, B and C**) because these cells, unlike bulk Tregs, underwent proliferation, entered the infected brain at even earlier times than M133 Tconv (**Figure 3D**), and decreased the number of pathogenic M133-specific CD4 T cells at this site (**Figure 8E**). Further, the majority of M133-specific Tregs, whether endogenous or transferred, expressed IL-10 in both the DCLN and brain [12] (**Figure 7** **and** **8G**). These results suggest a potential role for virus epitope-specific Tregs as a therapeutic option in encephalitis and perhaps in other infections. Compared to bulk Tregs, virus epitope-specific Tregs would have the advantage of specifically diminishing numbers and function of pathogenic CD4 T cells responding to the same epitope without generally suppressing the anti-virus T cell response. Thus far, Tregs have been used clinically in patients with autoimmune disease and transplantation (e.g., [35], [36], but our results suggest a possible role in viral infections.

## Materials and Methods

### Mice

Specific pathogen-free 6 week old C57BL/6 (B6) and Thy1.1 and CD45.1 congenic mice were purchased from the National Cancer Institute. Mice transgenic for the expression of an M133-specific public T cell receptor (M133 Tg mice) were developed as previously described [23]. Foxp3^gfp^ (B6.Cg-*FOXP3^tm2tch^*/J) mice, in which eGFP is expressed behind an IRES element, were purchased from Jackson Laboratories. Male mice were used in all experiments. Mice were maintained in the animal care facility at the University of Iowa.

### Virus infection

A neuroattenuated variant of the JHMV strain of MHV, rJ2.2 (a recombinant version of the J2.2-V-1 virus) [37] and a mutated rJ2.2 (rJ2.2.M_Y135Q_) in which the M133 epitope was engineered to abolish binding to the I-A^b^ antigen [38], were propagated in mouse 17Cl-1 cells and titered on HeLa-MHVR cells [39]. 6–7-week-old mice were inoculated intracerebrally with 600 PFU rJ2.2 or 2,000 PFU rJ2.2.MY135Q in 30 µl DMEM. After viral inoculation, mice were examined and weighed daily.

### Antibodies and flow cytometry

The following antibodies and streptavidin, purchased from BD Biosciences, eBiosciences and BioLegend, were used in this study: purified functional anti-CD3 (145-2C11) and anti-CD28 (37.51), B220-APC (RA3-6B2), CD4-FITC, -PE, -PerCP-Cy5.5, -PE-CY7, or -eFluor 450 (RM 4–5), CD8-APC (53–6.7), CD11b-APC or -eFluor 450 (M1/70), CD11c-PE (HL3), CD16/CD32-biotin (93), CD25-PE (PC61), CD45- PE (30-F11), CD69-PE (H1.2F3), CTLA-4-PE (UC10-4B9), CXCR3-PE or –APC (CXCR3-173), Foxp3-FITC, -PE, or -Alexa Fluor 647 (FJK-16s), I-A/I-E-PerCP-Cy5.5 (M5/114.15.2), ICOS-PE (7E.17G9), IFN-γ-PE or -APC (XMG1.2), IL-10-APC (JES5-16E3), T-bet-PE (eBio4B10), Thy1.1-PerCP-CY5.5 (OX-7), Thy1.2-PE or –APC (30-H12), streptavidin-APC, -eFluor 450 or -BV510. A PE Annexin V Apoptosis Detection Kit I was purchased from BD Pharmingen. Cells were analyzed using a FACSVerse or LSRII (BD).

### Isolation of mononuclear cells from brains

Brains harvested after PBS perfusion were dispersed and digested with 1 mg/ml collagenase D (Roche) and 0.1 mg/ml DNase I (Roche) at 37°C for 30 min. Dissociated brain was passed through a 70-µm cell strainer, followed by Percoll gradient (70/37%) centrifugation. Mononuclear cells were collected from the interphase, washed, and resuspended in culture medium for further analysis.

### Blood MHC class II tetramer staining

Peripheral blood was collected from the orbital sinus of WT-Foxp3^gfp^ or M133 Tg-Foxp3^gfp^ mice using heparinized Natelson blood collecting tubes (Fisher Scientific, Pittsburgh, PA). 100 µl blood were incubated with equal volumes of RP10 media containing 5U heparin and 1.6 µg PE-conjugated I-A^b^/M133 tetramers (obtained from the NIH/NIAID MHC Tetramer Core Facility, Atlanta, GA) for 1 hour at 37°C. Cells were then stained with anti-CD4-PerCP-Cy5.5 at 4°C, and red blood cells were removed with ACK lysing buffer.

### Intracellular cytokine and transcription factor staining

To detect IFN-γ and IL-10 production by antigen-specific CD4 effector T cells (Tconv) and Tregs, mononuclear cells isolated from the LN or brain were stimulated with 5 µM peptides M133 or S358 (CD4 T cells) or 1 µM S510 (CD8 T cells) (Bio-synthesis, Inc, Lewiston, TX) in complete RPMI 1640 medium for 6 h at 37°C, in the presence of 1 µl/ml Golgiplug (BD) and antigen-presenting cells (CHB3 cells, B cell line, H-2D^b^, I-A^b^). The rJ2.2-specific epitopes encompass residues 133–147 of the transmembrane (M) protein and residues 358–372 and 510–518 of the surface (S) glycoprotein [40], [41]. Intracellular expression of IFN-γ and IL-10 was assayed. A Foxp3 Staining Buffer Set (eBioscience) was used for Foxp3 or T-bet staining or when cells were analyzed for Foxp3 and cytokine expression simultaneously; otherwise, BD Cytofix/Cytoperm and Perm/Wash buffers (BD Biosciences) were used in intracellular cytokine staining assays.

### Adoptive transfer

For all adoptive transfers, lymphocytes were prepared from spleens and lymph nodes of donor mice. For transfer of bulk Tconv and Tregs, cells were purified from *Foxp3^gfp^*/Thy1.1 or *Foxp3^gfp^*/CD45.1 mice. For transfer of M133 Tconv and Tregs, donor M133 TCR Tg/*Foxp3^gfp^* mice were generated by crossing M133 TCR Tg mice with *Foxp3^gfp^* or *Foxp3^gfp^*/Thy1.1 mice. CD4^+^ T cells were negatively selected using a CD4 T Cell Isolation Kit II (containing a biotinylated antibody cocktail) and an AutoMACS (Miltenyi Biotech). Enriched CD4 T cells were further labeled with anti-mouse CD8-APC, B220-APC and streptavidin-APC. Tconvs (APC^−^GFP^−^) or Tregs (APC^−^GFP^hi^) were then sorted twice using yield mode followed by purity mode on a FACSDiva or FACSAria (BD). Cell purities were typically 99.4%–99.7%. Cells were then labeled with 3 µM CFSE (Invitrogen) or Violet dye (CellTrace Violet Cell Proliferation Kit, Invitrogen). Because about 50% and 97% of Tregs and Tconvs in M133 TCR Tg/*Foxp3^gfp^* mice were M133-specific (**Figure 1A**), Tregs and Tconvs were mixed at a 2∶1 ratio (1.5×10^5^ and 7.5×10^4^) in most co-transfer experiments to achieve a 1∶1 ratio of M133 Tregs to Tconv. Cells were transferred to 6–7 week congenic B6 mice 24 hours prior to rJ2.2 infection. The expansion index (fold-expansion of the overall culture) was obtained using FlowJo software, proliferation platform (Tree Star). In some experiments, recipient mice were treated intraperitoneally with 80 µg FTY720 (Cayman Chemical, Ann Arbor, MI) 0.5 hour prior to infection and once a day thereafter.

### *In vitro* suppression assay

B6 (Thy1.2) mice were infected with rJ2.2 or rJ2.2.M_Y135Q_ at day -8. To obtain responder cells, CD4 and CD8 T cells were sorted from the brains of rJ2.2-infected (M133-specific CD4) or of rJ2.2.M_Y135Q_-infected (S358-specific CD4 and S510-specific CD8) mice at day 0. At day -1, M133 Tregs were sorted from naïve M133 TCR Tg/*Foxp3^gfp^*/Thy1.1 mice as described above, stimulated with coated anti-CD3 mAb+anti-CD28 mAb (5 µg/ml each) for 24 hours and washed thoroughly before use. To set up the suppression assay (**Figure 9A**), 5×10^4^ Violet-labeled (2 µM) responders were co-cultured with 2.5×10^5^ irradiated CHB3 cells (2000 rad) and the indicated number of M133 Tregs per well, in the presence of 5 µM M133 peptide (for M133-specific CD4) or 5 µM S358+5 µM M133 peptide (for S358-specific CD4) or 1 µM S510+5 µM M133 peptide (for S510-specific CD8), in a 96-well round bottom plate. After 66 hours, cells were harvested and stained with anti-Thy1.2 and anti-CD4 or anti-CD8 antibodies. CD4 or CD8 responders were analyzed for Violet dye dilution by flow cytometry. The Division Index (DI, the average number of cell divisions) was obtained using FlowJo software (Tree Star, Inc.). The percentage of suppression by Tregs was calculated as follows: % suppression = 100% x [1−(DI of responders plus Tregs/DI of responders only)].

### Statistical analysis

Data are expressed as mean ± SEM. Two-tailed, unpaired Student *t* tests were used to analyze differences in mean values between groups in most experiments. Log-rank (Mantel-Cox) tests were used to analyze differences in survival. P values<0.05 were considered significant. *P<0.05, **P<0.01, ***P<0.001.

#### Ethics statement

This study was carried out in strict accordance with the recommendations in the Guide for the Care and Use of Laboratory Animals of the National Institutes of Health. Animal experiments were approved by the Institutional Animal Care and Use Committee at the University of Iowa (Protocol #1307138).
